# Olfactory drug delivery of artemether-curcumin combination for management of cerebral malaria

**DOI:** 10.1186/1475-2875-11-S1-P51

**Published:** 2012-10-15

**Authors:** Kunal Jain, Kuppusamy Gowthamarajan, Sumeet Sood, Kannan Elango, Bhorjraj Suresh

**Affiliations:** 1 Department of Pharmaceutics, J.S.S. College of Pharmacy (Constituent College of J.S.S. University, Mysore), Ootacamund, Tamilnadu-643001, India; 2 Department of Pharmacology, J.S.S. College of Pharmacy (Constituent College of J.S.S. University, Mysore), Ootacamund, Tamilnadu-643001, India; 3 J.S.S. University, Mysore, Karnataka-570015, India

## 

The objective of the present investigation was to explore the potential of nanoemulsion (NE) containing artemether-curcumin combination to accomplish the delivery of drugs to the brain via olfactory delivery system for management of cerebral malaria. The components for curcumin NE (C-NE) and artemether NE (A-NE) formulations were selected based on saturation solubility studies and were prepared by aqueous titration technique [1]. The mucoadhesive drug loaded nanoemulsions were made by adding chitosan (0.50% w/w) and stirred for 1 h. The nanoemulsions showed average globule size ranging from 32-70nm with polydispersity index of 0.221-1.000 and zeta potential in range of -12 to -28 mV. The formulations were subjected to thermodynamic stability tests like Heating-Cooling cycle, Freeze Thaw cycle and Centrifugation. All those formulations which passed the tests were subjected for further evaluation like transmittance, refractive index and electroconductivity tests. The percent transmittance of the formulations was >98% and refractive index was in range of 1.3-1.4 indicating transparent nature of nanoemulsions. Nanoemulsions exhibited high electroconductivity confirming that they were oil-in-water type. Transmission electron microscopy studies confirmed that globules were spherical in shape and size was in agreement with results obtained from globule size analysis. The nanoemulsions were evaluated for cytotoxicity studies using Vero cell lines by MTT assay. The CTC_50_ value of C-NE and A-NE was found to be 1950 meg/ ml and 2000mcg/ml respectively. Hemolytic activity was within the acceptable range revealing low toxicity risk of nanoemulsion. The *ex vivo* release studies of the formulated nanoemulsions were carried out using sheep nasal mucosa in comparison with drug suspension in simulated nasal fluid. The results revealed that release rate was biphasic and faster from drug suspension as compared to nanoemulsions. It can be concluded that nanoemulsions were prepared successfully having low particle size and can be used to deliver curcumin and artemether intranasally for management of cerebral malaria. The efficacy of the developed formulations either alone or in combination was evaluated further for antimalarial efficacy in *Plasmodium berghei* ANKA murine model of cerebral malaria in comparison with pure drug suspension administered intranasally and intravenously and showed promising results.
